# Rapid one-step CRISPR-cas vector assembly by isothermal spacer removal linearization and sequence-ligation independent cloning (ISRL-SLIC)^☆^^☆☆^

**DOI:** 10.1016/j.mex.2025.103567

**Published:** 2025-08-15

**Authors:** Claus Krogh Madsen, Tobias Hanak, Henrik Aronsson, Henrik Brinch-Pedersen

**Affiliations:** a Crop Genetics and Biotechnology, AU Flakkebjerg, Department of Agroecology, Aarhus University, Forsogsvej 1 4200 Slagelse, Denmark; b Department of Biological and Environmental Sciences, University of Gothenburg, Box 461 405 30 Gothenburg, Sweden

**Keywords:** New genomic techniques, Protospacer, Single strand annealing, Sequence independent, Ligation independent, CRISPR-Cas

## Abstract

CRISPR-Cas genome editing is a powerful tool in various fields, but current cloning methods can be time-consuming due to the frequent use of intermediate entry vectors and multiple steps involving restriction enzymes and ligases. These multiple steps can create a bottleneck in CRISPR-Cas experiments. In response to this challenge, we propose a highly efficient streamlined approach, which enables simultaneous linearization of the acceptor plasmid and protospacer cloning in a single isothermal reaction. This eliminates the need for entry vectors, pre-linearization of vectors, and *in vitro* ligation, thus significantly simplifying the cloning process. The method can be applied to clone short synthetic oligos for single protospacer constructs or multiple amplicons for multiplex genome editing designs. Either way, researchers can proceed directly to *Escherichia coli* transformation after a one-hour isothermal reaction and recover the final construct within two days. By combining the advantages of sequence-ligation independent cloning (SLIC) cloning with a streamlined workflow, our approach facilitates rapid and efficient construction of CRISPR-Cas vectors and holds the promise of accelerating research and development in genome editing and related fields.

To expedite the cloning of constructs, we propose a rapid one-step CRISPR-Cas vector assembly method that combines isothermal spacer removal with a sequence-ligation-independent cloning reaction.

We could show that **I**sothermal **S**pacer **R**emoval **L**inearization and **S**equence-**L**igation **I**ndependent **C**loning (ISRL-SLIC) can create single, double and triple protospacer constructs in one reaction with scalability.

The ISRL-SLIC reaction delivers clones under a broad range of oligo concentration making it a robust and time saving alternative to other methods for constructing CRISPR-Cas vectors.

## Graphical abstract

**Figure:**
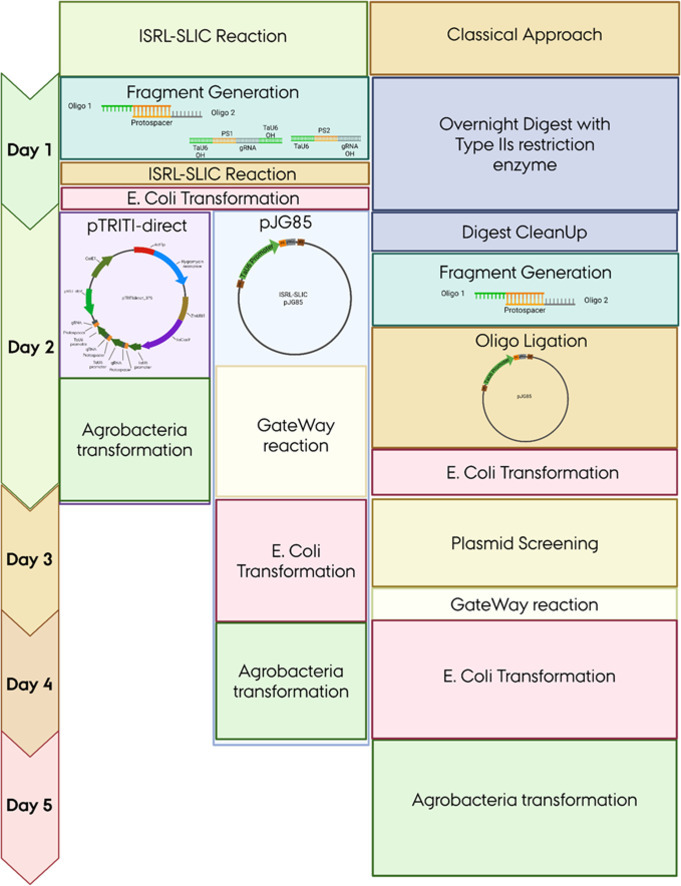

Created in BioRender. Hanak, T. (2025) https://BioRender.com/mr6ckw7Specifications table

**Table:** 

**Subject area**	Biochemistry, Genetics and Molecular Biology
**More specific subject area**	Cloning of CRISPR/Cas9 transformation vector
**Name of your method**	Isothermal Spacer Removal Linearization and Sequence-Ligation Independent Cloning (ISRL-SLIC)
**Name and reference of original method**	
**Resource availability**	Sequence Files are provided in the supplement, plasmids can be achieved by asking

## Background

CRISPR-Cas genome editing has become a tool of immense importance in diverse fields such as cell biology, oncology, microbiology and plant biotechnology. Generally, a CRISPR-Cas experiment starts with cloning an approximately 20-base pair synthetic oligonucleotide, the protospacer, in a plasmid vector. The protospacer must be cloned seamlessly between a promoter of choice and the conserved guide RNA (gRNA) sequence. Two prominent systems for cloning plant transformation vectors are the pANIC and the pTrans system [1,5,9]. The pANIC system is derived from a broad set of plant overexpression plasmids and adapted for CRISPR-Cas9 experiments in monocotyledonous plants by the addition of a protospacer entry plasmid (pJG85) and a Cas9 cassette donor plasmid (pJG80). The system uses Gateway cloning for final assembly of the constructs [5,9]. Gateway cloning utilises a two-step process involving the initial cloning of entry vectors and subsequent LR reactions to transfer the inserts into the destination vector (pANIC), which makes it a robust but time-consuming cloning system [[11], [12]]. The pTrans system utilizes a Golden Gate approach facilitated by Type IIs restriction enzymes, which cleave DNA outside of their recognition site, to generate the inserts for the destination vector [1,2]. However, both systems have the drawback of having to clone the protospacer in entry vectors before assembling the final plant transformation vector. The advent of single-strand annealing (SSA) and sequence and ligation-independent (SLIC) cloning approaches such as Gibson assembly, USER-Fusion and In-Fusion® have opened new possibilities for molecular cloning approaches [3,4,6]. These techniques rely on generating and annealing complementary single-stranded nucleotide termini to join nucleotides [4,13]. Ligation may be performed *in vitro* (for SSA) or in a host organism following transformation with the annealed assembly (SLIC) [4,13]. In-Fusion cloning relies on the vaccinia DNA polymerase with 3′−5′ exonuclease activity to generate single-stranded termini, promote their annealing, and fill out gaps [6,13]. This is achieved in an isothermal reaction, with incubation times typically between 5 and 60 min [13]. At this point, the user can proceed immediately to *Escherichia coli* transformation. SLIC cloning can be used for seamless cloning in a vector following the removal of a type IIS or type IIB restriction site flanked spacer (Spacer Removal Linearization, SRL) [8]. We hypothesized that SLIC cloning, here exemplified by In-Fusion® cloning would be suitable for simplified protospacer cloning. We were able to optimize the workflow, integrating SRL and SLIC in a single isothermal reaction; Isothermal Spacer Removal Linearization and Sequence-Ligation Independent Cloning (ISRL-SLIC). The method is fast, efficient and has a high degree of fidelity.

## Method details

### Genetic material

All constructs were derived from the plant CRISPR-Cas9 system previously reported [7] and pUC18 [10]. All oligos used in this work are listed in Table 1.

**Table 1:** Overview of oligos used in this study.

Primer name	Oligo sequence
ccdB_pJG85_Fw	TTGCTGCATCAGACTTGGAGACGGCGGCCGCATTAGGCACCCC
ccdB_pJG85_Rv	TGCTATTTCTAGCTCTAAAACGAGACGGTCGACCTGCAGACTGGC
AmpR_BspQI_Fw	TTGCTGCATCAGACTTGGAAGAGCGGTTTCTTAGACGTCAGGT
AmpR_Rv	TTACCAATGCTTAATCAGTGAGG
ccdBfusAmp_Fw	CTGATTAAGCATTGGTAAAGATCTGGATCCGGCTTA
ccdB_pJG85_BspQI_Rv	TGCTATTTCTAGCTCTAAAACGAAGAGCGTCGACCTGCAGACTGGC
pANIC_delBspQI_Fw	CTGGCCAGCGCACAGCCGATCAGCTGCAA
pANIC_delBspQI_Rv	GTCAGTGAGCGAGGAAGCGGTTGAGCGCCT
Cas9_NheI_Fw	GTGGTGGACAAGGGCG
Cas9_S_TCC_Rv	GAAGCCATCGGACTTCAGGAA
Cas9_S_TCC_Fw	TTCCTGAAGTCCGATGGCTTC
Cas9_PstI_Rv	CAGGTAGAGCTTCTCGTTCTG
TaU6_Fw_IF_EcoRICas	GCTTCCGGAAAGGGCGAATTGTTCCCTACTCTCGCGTT
gRNA_Rv_IFSpeI	GCAGGACTCTAGGGACTAGTGTCGACCTGCAGAAAAAAAGC
ZmUbiNcoI_Fw	TCTAGATCGGCGTTCCGGTC
ZmUbiEcoRI_Rv	GCCATGGTGAAGGGCGAATT
pANICpatch_Fw	CTTAATTAAGATATCGAGCTCGG
pANICpatch_Rv	CCCGGTACAAACCTGCAG
pANIC_NewMCS_Fw	TCGGCGTTAATTCAGCTGCAGCCGCGGACGCGTGATATC
pANIC_NewMCS_Rv	GATCGGGGAAATTCGAGCTCGATATCACGCGTCCGCGG
ModelPS_Fw	CTTGCTGCATCAGACTTCTCGAGGCGGCCGCGAGCTC
ModelPS_Rv	AACTTGCTATTTCTAGCTCTAAAACGAGCTCGCGGCCGCCTCGAG
Fragment1_PS1_fwd	TGCTGCATCAGACTTGGTAACGTGAATTCACCGGATGTTTTAGAGCTAGAA
gRNA_rev_MultiPS	ATAACGGGCTTGGTCAAAAAAAGCACCGACTCGGT
TaU6_Fwd_MultiPS	ACCGAGTCGGTGCTTTTTTTGACCAAGCCCGTTAT
TaU6_ps2_rev	CTAGACATCGGATCCGGGTCCCAAGTCTGATGCAGCA
PS2_gRNA_fwd	GGGACCCGGATCCGATGTCTAGTTTTAGAGCTAGAA
TaU6_PS3_rev	TTCTAGCTCTAAAACTCGGCAGAAGCTTTATTGTCCAAGTCTGATGCAGCA

### Biological material

Two different *E. coli* strains were used for cloning. The pJG85_*ccd*B and modified pANIC variants were generated and transformed into One Shot™ *ccd*B Survival™ 2 T1^R^ Competent Cells from Invitrogen™. The generated final pANIC plasmids containing the fully assembled protospacer inside were cloned into the Stellar™ competent cells from TaKaRa.

### Preparation of pJG85 variants with *ccd*B selection

For cloning of the pJG85_BsmBI_*ccd*B, we amplified the *ccd*B cassette from the pANIC_6A vector with the primers *ccdB*_pJG85_Fw/Rv (Table 1). Using In-Fusion® cloning, we ligated the amplified product with prepared pJG85, linearised with BsmBI, according to the manufacturer's instructions. The In-Fusion® reaction was then transformed into *E. coli* and recovered on chloramphenicol selection. A similar approach was used for the alternative variant of pJG85 containing the BspQI_*ccd*B cassette. The primers used for amplifying the ampicillin resistance cassette from pUC18 (AmpR_BspQI_Fw and AmpR_Rv) and for amplifying the *ccd*B cassette from pANIC_6A (ccdBfusAmp_Fw and ccdB_pJG85_BspQI_Rv) are listed in Table 1. The two amplicons were cloned into prepared pJG85 as described above and transformed accordingly. Clones were selected with LB-agar plates containing ampicillin.

### Preparation pTRITIdirect

To create a vector for direct cloning of protospacers in a binary *Agrobacterium* plasmid, BspQI recognition sites in pANIC_6A and pJG80 were deleted using site-directed mutagenesis. pANIC_6A was digested with BspQI, and the excised fragment was replaced by the amplicon of pANIC_delBspQI_Fw/Rv (Table 1) by In-Fusion® cloning. Clones were recovered on kanamycin selection. Likewise, pJG80 was digested with NheI and PstI. The excised fragment was replaced by the products of Cas9_NheI_Fw / Cas9_S_TCC_Rv and Cas9_S_TCC_Fw / Cas9_S_TCC_Rv (Table 1). Transformed *E. coli* (Stellar™) were selected using streptomycin. To assemble the vector, a Gateway reaction was performed according to the supplier’s manual (Invitrogen). The LR reaction contained pANIC_6a_ΔBspQI, pJG80_ΔBspQI and pJG85_BspQI_*ccd*B. The LR reaction was transformed into *E. coli* and recovered on LB-agar plates containing kanamycin and ampicillin. This strategy did not yield any correctly assembled clones after three attempts.

A smaller variant of pANIC_6A without the red fluorescent protein (RFP) cassette was prepared by In-Fusion® cloning the 11,539 bp SacI and 2707 bp PstI Fragments of pANIC_6A with the amplicons of pANICpatch_Fw/Rv (Table 1), template pANIC_6A and pANIC_NewMCS_Fw/Rv (Table 1), and no template. The resulting plasmid pANICΔRFP has additional unique restriction sites, MluI and SacII, as well as an additional EcoRV site in place of the RFP cassette. As described above for pANIC_6A, the two BspQI sites were also removed for pANIC_6AΔRFP to create pANICΔRFPΔBspQI. Gateway assembly using pANICΔRFPΔBspQI also failed. As an alternative strategy, we assembled the desired construct using In-Fusion® cloning.

**Fig. 1:**
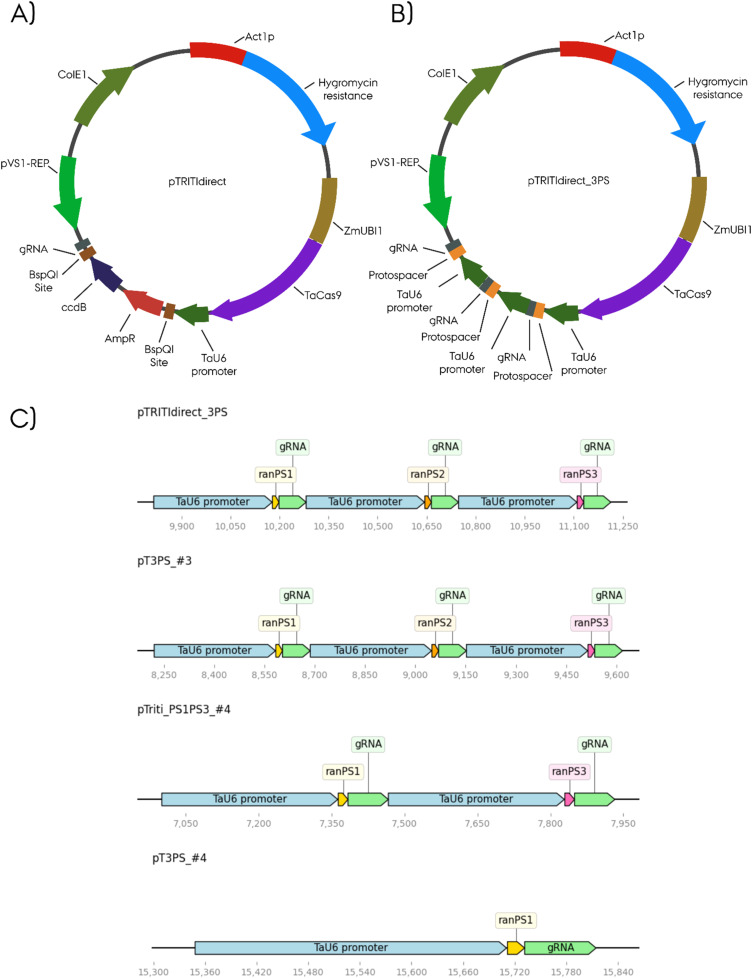
Overview of entry vector and assembled vector with sequence alignment of sequenced plasmids below. **A)** Vector map of the pTRITIdirect entry plasmid used for isothermal reaction. **B)** Vector map of the pTRITI_PS1PS2PS3 triple construct, showing the correct assembled plasmid with three protospacers inside. **C)** Graphical sequence alignment overview highlighting the tested plasmid's protospacer harboring region. A *in silico* variant pTRITI_direct3PS was used as a template for the alignment. The TaU6_promoter is shown in tortoise; the protospacer are in yellow, and the gRNAs are indicated in grey. Missing bases are highlighted in red on the sequence axis. Created in BioRender. Hanak, T. (2025) https://BioRender.com/kk5xhvg.

To this end, pANICΔRFPΔBspQI was digested with NcoI and SpeI and the large fragment was isolated. Cas9 was excised from pJG80ΔBspQI by EcoRI digest. The gRNA cassette with the ccdB spacer was amplified from pJG85_BspQI_*ccd*B with primers TaU6_Fw_IF_EcoRICas and gRNA_Rv_IFSpeI, and the missing part of the ZmUbi promoter was amplified with primers ZmUbiNcoI_Fw and ZmUbiEcoRI_Rv from a construct previously assembled by Gateway cloning. The resulting pTRITIdirect is essentially identical to the expected outcome of the unsuccessful Gateway cloning (Fig. 1).

### ISRL-SLIC cloning of protospacers

We prepared **I**sothermal **S**pacer **R**emoval - SLIC (ISRL-SLIC) reactions to generate the final vectors containing the designed protospacer. Each ISRL-SLIC reaction consisted of 100 ng appropriate plasmid, 3 µL of each oligo, 1 u (in 1 µL) restriction enzyme and 2 µL In-Fusion® 5x master mix to a final volume of 10 µL. The reaction was performed by incubation at 50 °C for one hour in a thermocycler with a heated lid. According to the manufacturer's instructions, one µL reaction was used to transform 30 µL competent *E. coli* cells. To test an In-Fusion® independent SLIC approach, we also used the NEBuilder HiFi DNA Assembly system. We used the same concentration of appropriate plasmid, protospacer and restriction enzyme, and added 2 µL of water in order to fulfil the manufacturer's volume requirements. We then proceeded with transformation into the delivered competent *E. coli* cells.

## Method validation

**Fig. 2:**
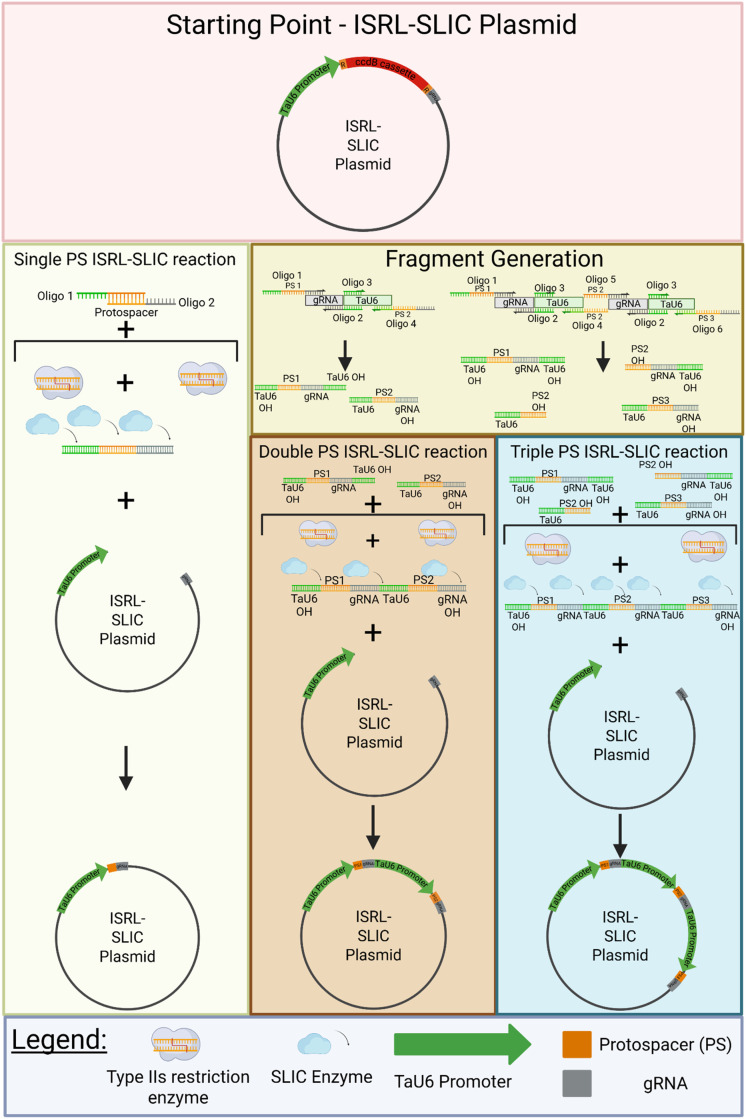
Procedure of ISRL-SLIC cloning. The ISRL-SLIC reaction can be used to integrate a single protospacer, or a combination of more protospacer directly into the target transformation vector, if suitable for this procedure. If more than a single protospacer is necessary, a PCR amplification of the fragments containing the TaU6, protospacer and gRNA is necessary. Created in BioRender. Hanak, T. (2025) https://BioRender.com/ugv0css.

**Fig. 3:**
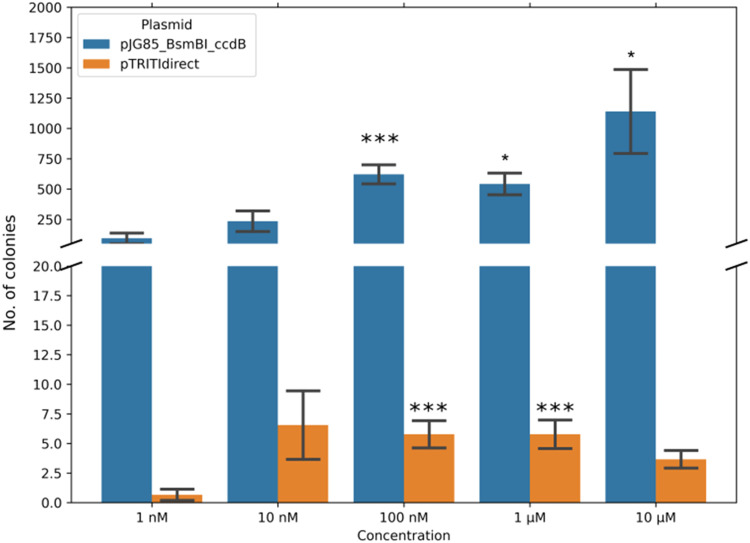
Visualisation of cloning efficiency based on colonies counted. Asterisks indicate a statistically significant difference from the 1 nM concentration. The bars show the mean number of colonies in the nine experiments evaluated. Error bars indicate the standard error of the mean. Control experiments showed no colonies forming and were therefore not visualized here.

For this investigation, we designed oligos for a model protospacer consisting of the restriction sites XhoI, NotI, and SacI, totalling 20 bp. The oligos contained overhangs of 17 bp (ModelPS_Fw) and 25 bp (ModelPS_Rv), which were complementary to the termini of Esp3I/BsmBI linearised pJG85, which contained the TaU6 promoter and the gRNA scaffold. The lengths of the overhangs were designed to exceed a calculated T_m_ of 50 °C. Additionally, we generated three additional model protospacers (Table 1), to test the insertion of multiple protospacers into the pTRITIdirect plasmid (Fig. 1). After designing the protospacer, we did a single protospacer reaction, and reactions with amplified fragments to clone two or three protospacers into the pTRITIdirect (Fig. 2). We were able to generate three different pTRITIdirect variants with either one, two or three model protospacer (Fig. 1+ Supplementary Material). We also wanted to verify the efficiency of our technique using different model protospacer concentrations. We prepared three independent model protospacer oligo dilution series from 1 nM to 10 µM, with the lowest concentration providing approximately 1:1 molar ratios for the plasmid and each oligo in the assembled reaction mixture. The colonies of each plate were counted, and significant differences are visualized with asterisks (*p* ≤ 0.05 *; *p* ≤ 0.01 **; *p* ≤ 0.005 ***) (Fig. 3).

The dilution series was used in the ISRL-SLIC reaction for the pTRITIdirect and the pJG85_BsmBI as two different plasmids to test. The colonies grown on the LB plates were then counted (Table 2).

**Table 2:** Descriptive statistics of colony count analysis for the pJG85_BsmBI_ccdB and pTRITIdirect ISRL-SLIC procedure.

Plasmid	Concentration	count	mean	std	min	25 %	50 %	75 %	max
pJG85_BsmBI_ccdB	1 nM	9	93.44444	131.4573	19	52	57	67	441
	10 nM	9	235	255.3101	57	88	139	267	875
	100 nM	9	621.8889	234.6681	307	493	591	724	988
	1 µM	9	542.5556	268.0658	152	354	425	793	965
	10 µM	9	1140.778	1038.378	6	11	939	2026	2538
pTRITIdirect	1 nM	9	0.666667	1.414214	0	0	0	0	4
	10 nM	9	6.555556	8.676277	0	1	2	11	26
	100 nM	9	5.777778	3.456074	2	4	5	6	13
	1 µM	9	5.777778	3.632416	2	3	5	9	12
	10 µM	9	3.666667	2.236068	0	2	3	5	7

The ISRL-SLIC produced colonies across a wide range of oligo concentrations (Fig. 3, Table 2). It is noteworthy, that for both plasmids, the lowest concentration (1 nM) yielded the least number of colonies. For the pJG85_BsmBI_ccdB plasmid, 10 µM produced the most colonies, while the best range for the pTRITIdirect was between 10 nM and 1 µM (Table 2, Fig. 3). We also attempted the ISRL-SLIC procedure in conjunction with the NEBuilder HiFi DNA Assembly. We successfully counted the colonies from a single experiment, demonstrating independence from the In-Fusion assembly (Supplementary Material).

Thirty colonies (six representing each oligo dilution) were picked for plasmid purification. The purified plasmids were digested with NotI, SacI and XhoI in separate reactions. Twenty-two of thirty clones showed the expected restriction pattern. The imperfect clones were distributed as two, two and four from 1 nM, 1 µM and 10 µM reactions, respectively. There were no imperfect clones with 10 and 100 nM concentrations (results not shown). All clones from the 1 nM and 10 µM set and the two imperfect clones from the 1 µM set were sequenced across the cloning site. The sequencing showed that all the clones were indeed correct, irrespective of the restriction pattern. This implies that reaction fidelity is high, and the efficiency has an optimum of approximately 100 nM or 100 times the molar excess of the oligos; however, it is also possible to obtain the desired clones when the concentration deviates two orders of magnitude from the optimum.

## Limitations

The efficiency of this method is dependent on two factors. Firstly, the number of protospacers can influence the outcome of your cloning experiments. With an increase in fragments, the efficiency can be reduced, however, up to 16 kBp and three fragments, a high efficiency should be reached [15]. Secondly, the plasmid size has a strong effect on the transformation efficiency; for instance, a smaller plasmid, such as pJG85_BsmBI_ccdB, which is around 5 kBp, can yield more colonies than a larger plasmid [14]. This, however, can increase the time required to create your transformation vector. Lastly, the concentration of oligos used in the assembly reaction mix influences the outcome. While we could achieve clones in a broad concentration range from 1 nM to 10 µM, the sweet spot lies around 100 nM according to our experiments.

## CRediT authorship contribution statement

**Claus Krogh Madsen:** Conceptualization, Methodology, Validation, Investigation, Writing – original draft, Writing – review & editing, Supervision. **Tobias Hanak:** Validation, Investigation, Writing – original draft, Writing – review & editing, Visualization, Software. **Henrik Aronsson:** Investigation, Writing – original draft, Writing – review & editing. **Henrik Brinch-Pedersen:** Writing – review & editing, Project administration, Funding acquisition.

## Declaration of competing interest

The authors declare that they have no known competing financial interests or personal relationships that could have appeared to influence the work reported in this paper.

## Data Availability

Data will be made available on request.

## Appendix Supplementary materials

Image, application 1

Image, application 2

Image, application 3

Image, application 4

Image, application 5

Supplementary material 1: GeneBank Files for plasmids used in this work. Image, application 6

Supplementary material 2: Alignment of positive clones. Image, application 7

Supplementary material 3: Colony counts of NEBuilder HiFi DNA Assembly Image, application 8
