# Supplementary material for: Rapid one-step CRISPR-cas vector assembly by isothermal spacer removal linearization and sequence-ligation independent cloning (ISRL-SLIC)
Source: MethodsX. 2025 Aug 15;15:103567. doi: 10.1016/j.mex.2025.103567 (PMC12395069; doi:10.1016/j.mex.2025.103567)
Supplement: Supplementary file 7 — Supplementary material 2: Alignment of positive clones. [file mmc7.pdf]

```
                  9817                                                                                                     9926
pTRITIdirec...  gaccaagcccgttattctgacagttctggtgctcaacacatttatatttatcaaggagcacattgttactcactgctaggagggaatcgaactaggaatattgatcagag
pT3PS_#3        gaccaagcccgttattctgacagttctggtgctcaacacatttatatttatcaaggagcacattgttactcactgctaggagggaatcgaactaggaatattgatcagag
pT3PS_#4        gaccaagcccgttattctgacagttctggtgctcaacacatttatatttatcaaggagcacattgttactcactgctaggagggaatcgaactaggaatattgatcagag
pTriti_PS1P...  gaccaagcccgttattctgacagttctggtgctcaacacatttatatttatcaaggagcacattgttactcactgctaggagggaatcgaactaggaatattgatcagag

..............................................................................................................................

                  9927                                                                                                    10036
pTRITIdirec...  gaactacgagagagctgaagataactgccctctagctctcactgatctgggtcgcatagtgagatgcagcccacgtgagttcagcaacggtctagcgctgggcttttagg
pT3PS_#3        gaactacgagagagctgaagataactgccctctagctctcactgatctgggtcgcatagtgagatgcagcccacgtgagttcagcaacggtctagcgctgggcttttagg
pT3PS_#4        gaactacgagagagctgaagataactgccctctagctctcactgatctgggtcgcatagtgagatgcagcccacgtgagttcagcaacggtctagcgctgggcttttagg
pTriti_PS1P...  gaactacgagagagctgaagataactgccctctagctctcactgatctgggtcgcatagtgagatgcagcccacgtgagttcagcaacggtctagcgctgggcttttagg

..............................................................................................................................

                  10037                                                                                                   10146
pTRITIdirec...  cccgcatgatcgggcttttgtcgggtggtcgacgtgttcacgattggggagagcaacgcagcagttcctcttagtttagtcccacctcgcctgtccagcagagttctgac
pT3PS_#3        cccgcatgatcgggcttttgtcgggtggtcgacgtgttcacgattggggagagcaacgcagcagttcctcttagtttagtcccacctcgcctgtccagcagagttctgac
pT3PS_#4        cccgcatgatcgggcttttgtcgggtggtcgacgtgttcacgattggggagagcaa------------------------------------------------------
pTriti_PS1P...  cccgcatgatcgggcttttgtcgggtggtcgacgtgttcacgattggggagagcaacgcagcagttcctcttagtttagtcccacctcgcctgtccagcagagttctgac

..............................................................................................................................

                  10147                                                                                                   10256
pTRITIdirec...  cggtttataaactcgcttgctgcatcagacttggtaacgtGAATTCaccggatgttttagagctagaaatagcaagttaaaataaggctagtccgttatcaacttgaaaa
pT3PS_#3        cggtttataaactcgcttgctgcatcagacttggtaacgtgaattcaccggatgttttagagctagaaatagcaagttaaaataaggctagtccgttatcaacttgaaaa
pT3PS_#4        --------------------------------------------------------------------------------------------------------------
pTriti_PS1P...  cggtttataaactcgcttgctgcatcagacttggtaacgtgaattcaccggatgttttagagctagaaatagcaagttaaaataaggctagtccgttatcaacttgaaaa

..............................................................................................................................

                  10257                                                                                                   10366
pTRITIdirec...  agtggcaccgagtcggtgctttttttgaccaagcccgttattctgacagttctggtgctcaacacatttatatttatcaaggagcacattgttactcactgctaggaggg
pT3PS_#3        agtggcaccgagtcggtgctttttttgaccaagcccgttattctgacagttctggtgctcaacacatttatatttatcaaggagcacattgttactcactgctaggaggg
pT3PS_#4        --------------------------------------------------------------------------------------------------------------
pTriti_PS1P...  agtggcaccgagtcggtgctttttttgaccaagcccgttattctgacagttctggtgctcaacacatttatatttatcaaggagcacattgttactcactgctaggaggg

..............................................................................................................................

                  10367                                                                                                   10476
pTRITIdirec...  aatcgaactaggaatattgatcagaggaactacgagagagctgaagataactgccctctagctctcactgatctgggtcgcatagtgagatgcagcccacgtgagttcag
pT3PS_#3        aatcgaactaggaatattgatcagaggaactacgagagagctgaagataactgccctctagctctcactgatctgggtcgcatagtgagatgcagcccacgtgagttcag
pT3PS_#4        --------------------------------------------------------------------------------------------------------------
pTriti_PS1P...  aatcgaactaggaatattgatcagaggaactacgagagagctgaagataactgccctctagctctcactgatctgggtcgcatagtgagatgcagcccacgtgagttcag

..............................................................................................................................
```

```
               10477                                                                                                   10586
pTRITIdirec... caacggtctagcgctgggcttttaggcccgcatgatcgggcttttgtcgggtggtcgacgtgttcacgattggggagagcaacgcagcagttcctcttagtttagtccca
pT3PS_#3       caacggtctagcgctgggcttttaggcccgcatgatcgggcttttgtcgggtggtcgacgtgttcacgattggggagagcaacgcagcagttcctcttagtttagtccca
pT3PS_#4       --------------------------------------------------------------------------------------------------------------
pTriti_PS1P... caacggtctagcgctgggcttttaggcccgcatgatcgggcttttgtcgggtggtcgacgtgttcacgattggggagagcaacgcagcagttcctcttagtttagtccca

.............................................................................................................................

               10587                                                                                                   10696
pTRITIdirec... cctcgcctgtccagcagagttctgaccggtttataaactcgcttgctgcatcagacttgggacccGGATCCgatgtctagttttagagctagaaatagcaagttaaaata
pT3PS_#3       cctcgcctgtccagcagagttctgaccggtttataaactcgcttgctgcatcagacttgggacccggatccgatgtctagttttagagctagaaatagcaagttaaaata
pT3PS_#4       --------------------------------------------------------------------------------------------------------------
pTriti_PS1P... cctcgcctgtc---------------------------------------------------------------------------------------------------

.............................................................................................................................

               10697                                                                                                   10806
pTRITIdirec... aggctagtccgttatcaacttgaaaaagtggcaccgagtcggtgctttttttgaccaagcccgttattctgacagttctggtgctcaacacatttatatttatcaaggag
pT3PS_#3       aggctagtccgttatcaacttgaaaaagtggcaccgagtcggtgctttttttgaccaagcccgttattctgacagttctggtgctcaacacatttatatttatcaaggag
pT3PS_#4       --------------------------------------------------------------------------------------------------------------
pTriti_PS1P... --------------------------------------------------------------------------------------------------------------

.............................................................................................................................

               10807                                                                                                   10916
pTRITIdirec... cacattgttactcactgctaggagggaatcgaactaggaatattgatcagaggaactacgagagagctgaagataactgccctctagctctcactgatctgggtcgcata
pT3PS_#3       cacattgttactcactgctaggagggaatcgaactaggaatattgatcagaggaactacgagagagctgaagataactgccctctagctctcactgatctgggtcgcata
pT3PS_#4       --------------------------------------------------------------------------------------------------------------
pTriti_PS1P... --------------------------------------------------------------------------------------------------------------

.............................................................................................................................

               10917                                                                                                   11026
pTRITIdirec... gtgagatgcagcccacgtgagttcagcaacggtctagcgctgggcttttaggcccgcatgatcgggcttttgtcgggtggtcgacgtgttcacgattggggagagcaacg
pT3PS_#3       gtgagatgcagcccacgtgagttcagcaacggtctagcgctgggcttttaggcccgcatgatcgggcttttgtcgggtggtcgacgtgttcacgattggggagagcaacg
pT3PS_#4       ------------------------------------------------------------------------------------------------------------cg
pTriti_PS1P... --------------------------------------------------------------------------------------------------------------

.............................................................................................................................

               11027                                                                                                   11136
pTRITIdirec... cagcagttcctcttagtttagtcccacctcgcctgtccagcagagttctgaccggtttataaactcgcttgctgcatcagacttggacaataAAGCTTctgccgagtttt
pT3PS_#3       cagcagttcctcttagtttagtcccacctcgcctgtccagcagagttctgaccggtttataaactcgcttgctgcatcagacttggacaataaagcttctgccgagtttt
pT3PS_#4       cagcagttcctcttagtttagtcccacctcgcctgtccagcagagttctgaccggtttataaactcgcttgctgcatcagacttggtaacgtgaattcaccggatgtttt
pTriti_PS1P... -------------------------------------cagcagagttctgaccggtttataaactcgcttgctgcatcagacttggacaataaagcttctgccgagtttt

.............................................................................................................................
```

```
               11137                                                                      11214
pTRITIdirec... agagctagaaatagcaagttaaaataaggctagtccgttatcaacttgaaaaagtggcaccgagtcggtgcttttttt
pT3PS_#3       agagctagaaatagcaagttaaaataaggctagtccgttatcaacttgaaaaagtggcaccgagtcggtgcttttttt
pT3PS_#4       agagctagaaatagcaagttaaaataaggctagtccgttatcaacttgaaaaagtggcaccgagtcggtgcttttttt
pTriti_PS1P... agagctagaaatagcaagttaaaataaggctagtccgttatcaacttgaaaaagtggcaccgagtcggtgcttttttt

..............................................................................................................................
```
